# Acute Bilateral Achilles Tendon Rupture in a Middle-Aged Patient: A Rare but Debilitating Injury

**DOI:** 10.7759/cureus.48716

**Published:** 2023-11-13

**Authors:** Efstathios Konstantinou, Antonios Koutalos, Vasileios Akrivos, Theodoros Mylonas, Sokratis Varitimidis

**Affiliations:** 1 Department of Orthopaedic Surgery & Musculoskeletal Trauma, University General Hospital of Larissa, Larissa, GRC

**Keywords:** achilles tendon rupture, bilateral, functional outcome, krackow technique, surgical treatment

## Abstract

Acute Achilles tendon (AT) rupture is an infrequent yet incapacitating injury that demands prompt diagnosis and effective intervention. While unilateral ruptures are more common, bilateral occurrences are exceedingly rare, particularly without predisposing factors. This case report presents an instance of a 52-year-old male patient who suffered a bilateral AT rupture during a soccer game. Physical examination and ultrasound confirmed bilateral AT tears, prompting surgical repair. The surgical procedure involved trimming the degenerated tendon ends, using a modified Krackow repair technique, and finally suturing the paratendon. Postoperatively, a tailored rehabilitation program was employed, encompassing bed-to-chair transfer for six weeks and partial weight-bearing afterward using Achilles braces. At the last follow-up, at nine months, the patient was able to walk bearing full weight with satisfactory clinical and functional outcomes. This report underscores the successful management of a rare case of bilateral acute AT rupture through surgical intervention and a tailored rehabilitation protocol. Bilateral AT ruptures necessitate an individualized approach, taking into account the complexities of simultaneous bilateral injuries.

## Introduction

The Achilles tendon (AT) is the thickest and strongest tendon of the human body, formed by the confluence of the medial and lateral gastrocnemius and soleus tendons [1]. Nevertheless, it is the most frequently ruptured lower limb tendon, with a prevalence of roughly 20% of all large tendon injuries [2]. Unilateral ruptures occur mostly in patients during their fourth and fifth decades when engaging in high-energy activities, predominantly in males [3]. Bilateral AT rupture is extremely rare, especially in the absence of predisposing factors or high-energy trauma [4]. A bilateral acute AT rupture is a particularly challenging injury, as it can result in significant disability and impede the ability to perform daily activities [5]. The management of this condition is complex and can vary depending on the patient’s age, medical history, and degree of injury.

This case report presents a rare case of a middle-aged patient who suffered from an abrupt bilateral AT rupture during a low-energy movement. The case highlights the importance of prompt diagnosis and proper management to minimize the impact of this debilitating injury and ensure a successful outcome. This article aims to provide insights into the diagnosis, management, and rehabilitation of bilateral acute AT rupture in middle-aged patients.

## Case presentation

A 52-year-old male patient presented to our emergency department, unable to walk or stand without support, with acute, severe pain in both ankles. This sudden pain started during a soccer game at the moment the patient needed to sprint. He did not mention any contact during the incident. His medical history was clear, except for a slightly elevated body mass index (BMI) of 27.5 kg/m^2^. Blood tests were not suggestive of any chronic rheumatologic or metabolic disease or drug use. He reported moderate weekly alcohol consumption, and he was a non-smoker. No use of fluoroquinolones or steroids was mentioned by the patient during the last few years.

After a thorough physical examination and X-rays of both lower limbs, a bilateral AT rupture was diagnosed. The Thompson “squeeze” test was positive, and there was an inability to plantar flex both ankles against resistance (Video 1).

**Video 1:** Preoperative examination: The patient is in the supine position before surgery. A positive Thompson “squeeze” test is performed bilaterally. Inadequate plantar flexion of the foot with a squeeze of the calf muscles

**Figure 1:**
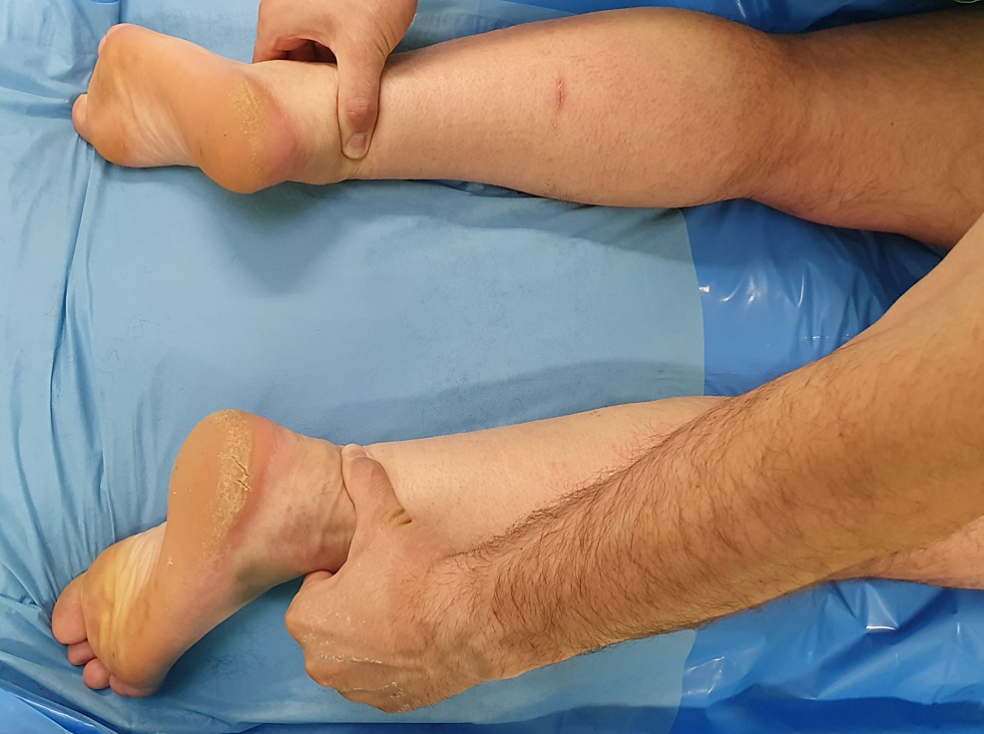
Preoperative examination: The patient is in the supine position before surgery. A palpable gap was evident 4 to 6 cm proximal to the insertion of the Achilles tendon.

In addition, a palpable gap was evident 4 to 6 cm proximal to the AT insertion bilaterally (Figure 1).

Due to the unavailability of a fast MRI scan to verify our diagnosis, we performed an ultrasound examination, confirming both AT’s complete tears. Achilles tendon Total Rupture Score (ATRS) [6] was 11 for the right and 15 for the left leg, and the ankle-hindfoot American Orthopedic Foot and Ankle Society Score (AOFAS) [7] score was calculated at 28 and 48, respectively. Open surgical repair of both ATs was proposed to the patient, who consented.

**Figure 2:**
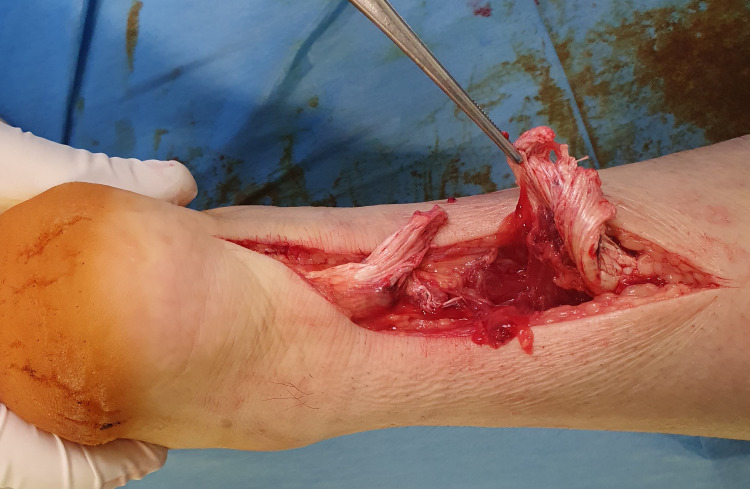
Achilles tendon rupture of the right foot; degeneration is noted on both ends of the tendon that needed partial excision before repair.

**Figure 3:**
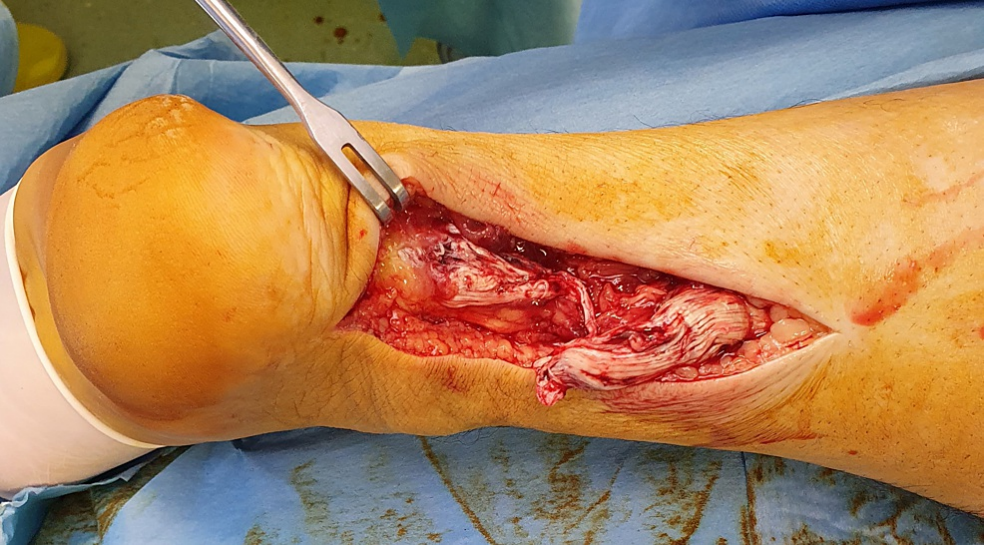
Achilles tendon rupture of the left foot; degeneration is noted on both ends of the tendon that needed partial excision before repair.

Under general anesthesia and in the prone position, the rupture of both ATs was again verified. During surgery, excessive degeneration was noted on both ATs (Figures 2, 3).

**Figure 4:**
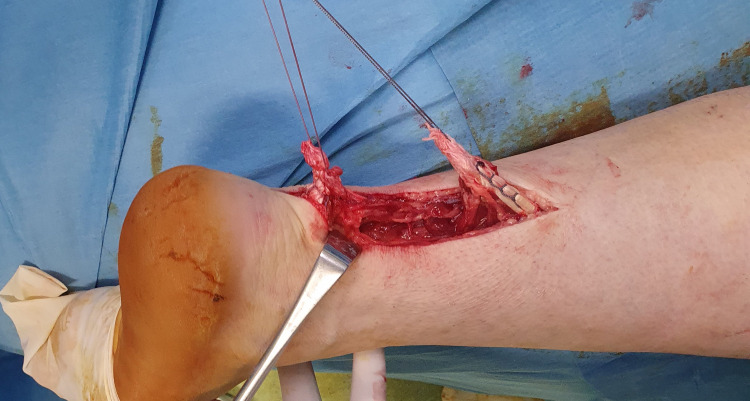
The Krakow technique: The two free ends of the tendon are secured with two non-absorbable sutures in a locking fashion.

**Figure 5:**
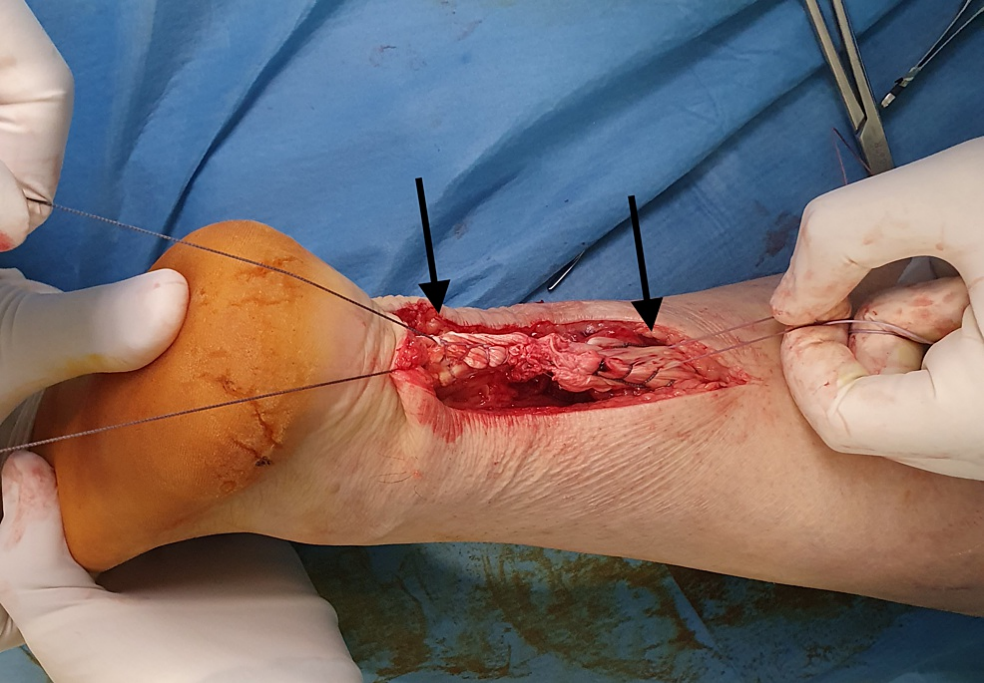
Modification of the Krakow technique: The free ends of one suture are passed peripherally to encircle the transverse limb of the opposite suture (black arrows).

**Figure 6:**
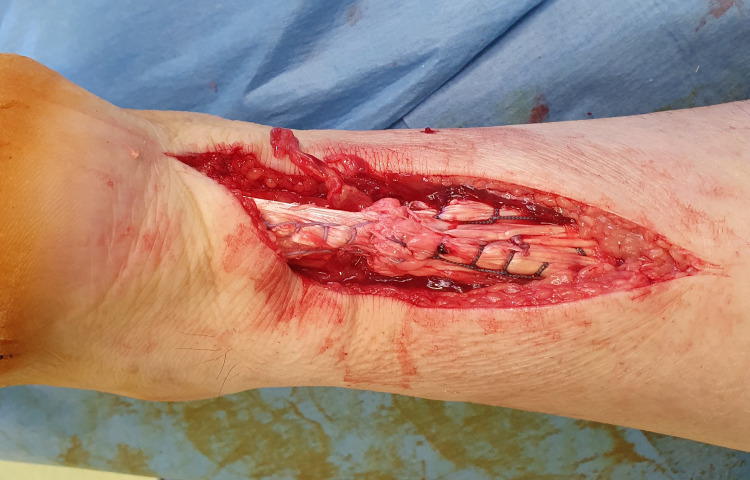
Repair of the right Achilles tendon; final result of the repair: A good approximation of the ends of the tendon is achieved. The final knot is not included at the rupture site.

**Figure 7:**
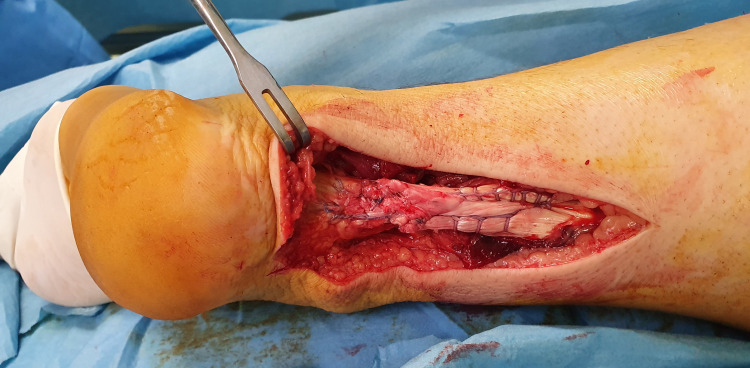
Repair of the right Achilles tendon; final result of the repair: A good approximation of the ends of the tendon is achieved. The final knot is not included at the rupture site.

Both AT ends were trimmed to normal tissue after removing 1 cm of abnormal tissue. Ethibond No. 5 sutures were used for the repair. A “giftbox” modification of the Krackow technique, as proposed by Labib et al. [8], was utilized in which the free ends of one suture are passed peripherally to encircle the transverse limb of the opposite suture (Figures 4-7).

Finally, the peritendons were sutured with a Vicryl No. 4-0 suture. After repair, satisfactory plantar flexion of the ankle was noted with a passive squeeze of the calf muscles (negative Thompson test) (Video 2).

**Video 2:** Repair of the right Achilles tendon: Plantar flexion with a squeeze of the calf muscles is restored.

No drains were placed in the wound, and a splint with the ankle in 30 degrees of plantarflexion was applied bilaterally. Low-molecular-weight heparin was prescribed for deep vein thrombosis prophylaxis for six weeks until mobilization of the patient.

The patient remained immobilized for six weeks, and only bed-to-chair transfers were allowed. At six weeks, the patient began to walk on Achilles braces in ankle equinus with the help of three boot wedges. The wedges were removed each week, and in the ninth week, the patient was walking on AT boots with 0 degrees of plantar flexion. Forceful passive ankle dorsiflexion was discouraged. The patient was weaned off the boots four months after the repair, and he was advised to use 1 cm heel lifts for another month. The rehabilitation was uneventful, and the patient was able to walk bearing full weight at the last follow-up at nine months. Dorsal flexion was measured at 10 degrees and plantar flexion at 40 bilaterally, while ATRS was 78 and 85 for the right and left legs, respectively (Videos 3, 4).

**Video 3:** Final clinical outcome of the right foot: Satisfactory active plantar and dorsal foot flexion is achieved at the last follow-up after nine months.

**Video 4:** Final clinical outcome of the left foot: Satisfactory active plantar and dorsal foot flexion is achieved at the last follow-up after nine months.

**Figure 8:**
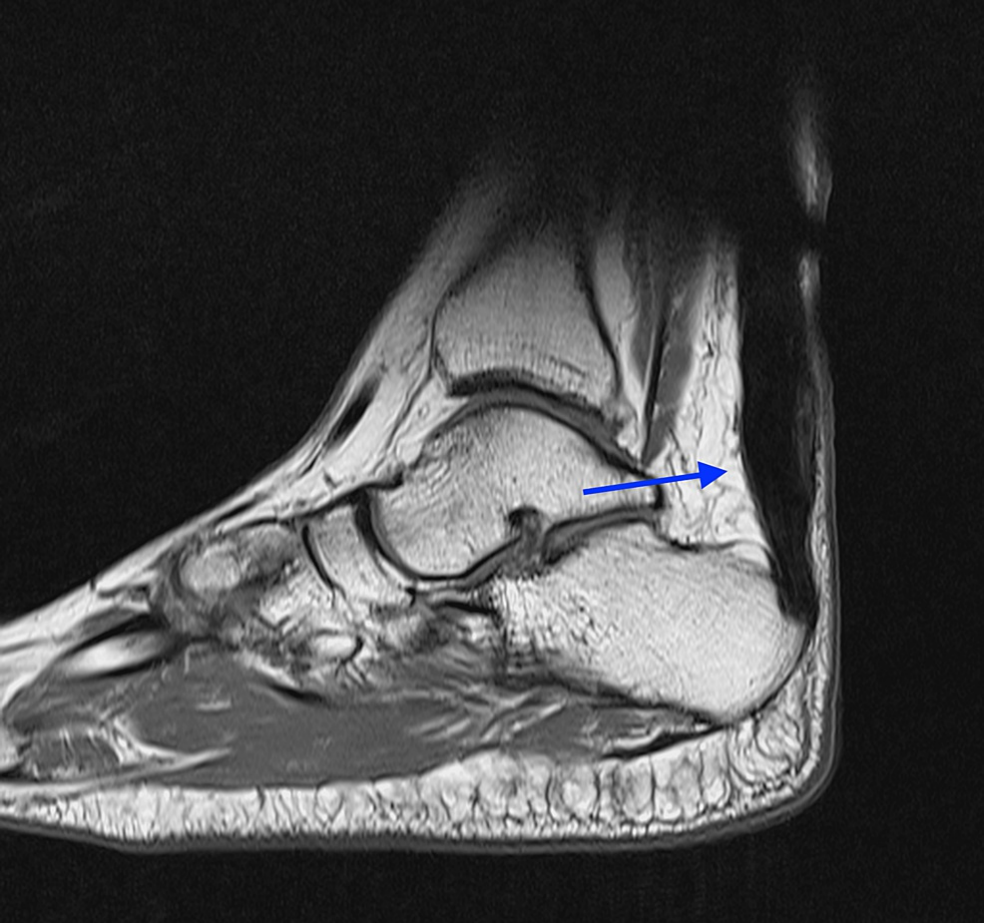
MRI of the right foot: A T1 weighted image depicting complete healing of the repair (blue arrow)

**Figure 9:**
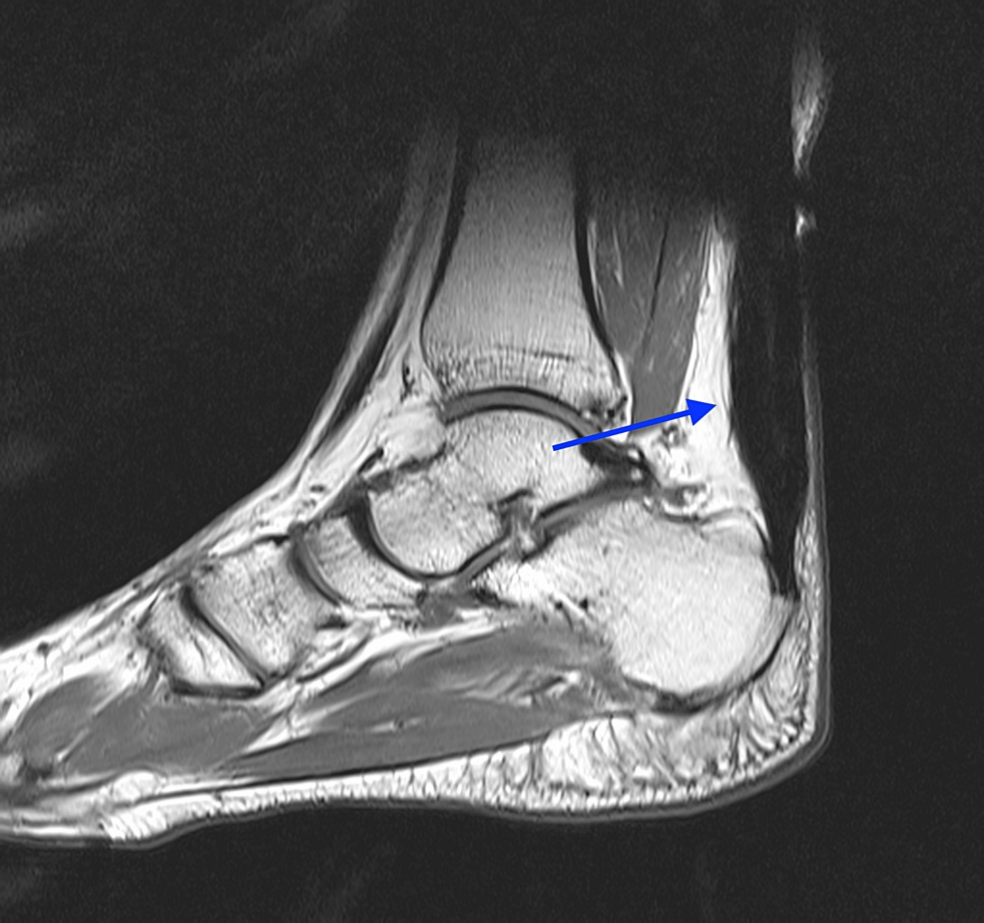
MRI of the left foot: A T1 weighted image depicting complete healing of the repair (blue arrow)

The AOFAS score was evaluated at 100 out of 100 for both feet. An MRI at six months confirmed the AT healing on both sides (Figures 8, 9).

## Discussion

Unilateral acute AT rupture most often occurs in healthy individuals in their thirties to fifties during highly energetic activities. Many patients report feeling a violent strike above the heel and instant pain in the back of the leg. There is no agreement in the literature on whether the rupture is due to degeneration and recurrent microtrauma or mechanical failure during an abrupt contraction [9,10].

Bilateral AT rupture occurs in only 1% of the patients with AT injury [5]. It is typically reported in older patients treated with steroids; however, there have been several other risk factors described [11]. These factors may include medication such as fluoroquinolones [12] or statins but also chronic diseases such as rheumatoid arthritis, systemic lupus erythematosus, chronic renal failure, and hyperparathyroidism [13]. Smoking has also been implemented as a major risk factor for AT rupture. Our patient did not have any of the above risk factors.

Controversy still surrounds the treatment of AT rupture, with recent studies stating that conservative treatment using specific protocols for rehabilitation has similar results regarding healing and functional outcomes as well as re-rupture rates compared with surgical repair, which is usually suggested in younger, more active patients [14]. The management of bilateral acute AT rupture differs from that of unilateral rupture, as it requires a more comprehensive approach and close coordination between the patient and the healthcare team. Bilateral AT ruptures can pose a significant challenge, as they can impair the ability to walk and perform daily activities, leading to increased disability and decreased quality of life. Open surgical management was chosen because anatomical reconstruction and greater suture strength were desirable, even if open repair is associated with higher complication rates involving wound healing problems, infections, and thrombosis [14]. Rehabilitation is slower, given the fact that both AT repairs need protection.

Clinical outcomes of unilateral AT rupture are generally favorable, with successful surgical repair resulting in complete restoration of function and resolution of symptoms in most patients [15]. However, the outcomes of bilateral ruptures are more variable, as they can be complicated by additional factors such as muscle weakness, gait abnormalities, and compensatory mechanisms. Kawtharani et al. reported satisfactory results after bilateral AT rupture due to ciprofloxacin use [16]. Similar results were also presented by Kapoor et al. after managing one partial AT and one complete rupture [17]. A personalized rehabilitation program provides the best functional outcome after operative management of bilateral AT rupture.

In our case, surgical repair of both ATs was performed, and weight-bearing and mobilization started six weeks postoperatively with the use of functional AT braces, leading to a satisfactory clinical outcome.

## Conclusions

A bilateral acute AT rupture is a rare but debilitating injury that necessitates prompt diagnosis and appropriate treatment. In this case report, we have demonstrated the successful outcome of surgical repair and rehabilitation in a middle-aged patient with a bilateral acute AT rupture. Recognizing the differences in management between unilateral and bilateral ruptures is crucial, as they can impact clinical outcomes. Further research is required to better understand the outcomes and the optimal management of bilateral acute AT ruptures.
